# Supplementary material for: Implementation of a Psychiatric Consultation for Healthcare Workers during First Wave of COVID-19 Outbreak
Source: Int J Environ Res Public Health. 2022 Apr 14;19(8):4780. doi: 10.3390/ijerph19084780 (PMC9025663; doi:10.3390/ijerph19084780)
Supplement: Supplementary file 1 [file ijerph-19-04780-s001.zip › ijerph-1616862-supplementary.pdf]

For use by BENZAKOUR Lamyae only. Received from Mind Garden, Inc. on December 17, 2021

Permission for BENZAKOUR Lamyae to reproduce 50 copies within three years of December 17, 2021

Maslach Burnout Inventory

Instruments and Scoring Guides

English: MBI-HSS, MBI-HSS (MP), MBI-ES, MBI-GS, and MBI-GS (S)

French: MBI-HSS and MBI-GS

Christina Maslach
Susan E. Jackson
Michael P. Leiter
Wilmar B. Schaufeli
Richard L. Schwab

Published by Mind Garden
info@mindgarden.com
www.mindgarden.com

Important Note To Licensee

If you have purchased a license to reproduce or administer a fixed number of copies of an existing Mind Garden instrument, manual, or workbook, you agree that it is your legal responsibility to compensate the copyright holder of this work — via payment to Mind Garden — for reproduction or administration in any medium. **Reproduction includes all forms of physical or electronic administration including online survey, handheld survey devices, etc.**

The copyright holder has agreed to grant a license to reproduce the specified number of copies of this document or instrument **within three years from the date of purchase.**

You agree that you or a person in your organization will be assigned to track the number of reproductions or administrations and will be responsible for compensating Mind Garden for any reproductions or administrations in excess of the number purchased.

This instrument is covered by U.S. and international copyright laws as well as various state and federal laws regarding data protection. Any use of this instrument, in whole or in part, is subject to such laws and is expressly prohibited by the copyright holder. If you would like to request permission to use or reproduce the instrument, in whole or in part, contact Mind Garden, Inc.

MBI-General Survey: Copyright ©1996 Wilmar B. Schaufeli, Michael P. Leiter, Christina Maslach & Susan E. Jackson.
MBI-Human Services Survey: Copyright ©1981 Christina Maslach & Susan E. Jackson.
MBI-Educators Survey: Copyright ©1986 Christina Maslach, Susan E. Jackson & Richard L. Schwab.
All rights reserved in all media. Published by Mind Garden, Inc., www.mindgarden.com

For use by BENZAKOUR Lamyae only. Received from Mind Garden, Inc. on December 17, 2021

Translation Quality not Guaranteed

Mind Garden instrument translations are of varying quality. Many are done by researchers. Some translations are translated and then back-translated to check the quality, while others have not been back-translated. We typically do not know the dialect of the translation. We also do not have validation data on the translation. Some translations do not include all of the items that are on the English form; sometimes a scale or scales are missing on the translation. You will receive what we have with no warranty or assurance of quality or dialect. Basically, we try to provide you with what we have available to facilitate your work.

For use by BENZAKOUR Lamyae only. Received from Mind Garden, Inc. on December 17, 2021

Note to Survey Administrators

Avoid Sensitization to Burnout. People have widely varying beliefs about burnout. To minimize the reactive effect of such personal beliefs or expectations, it is important that respondents be unaware that the MBI is a burnout measure and that they not be sensitized to the general issue of burnout. For this reason, the labels used on the survey do not include the word “burnout”. You will often see survey administrators use one of the following acceptable terms or similar wording on the survey or referring to it:

- MBI Assessment
- Wellness Survey
- Job Attitudes Assessment
- Employee Well-Being Survey

The scale should be presented as a survey of job-related attitudes and not be linked to burnout in any way. Of course, once the measure has been administered to all respondents, then an explanation about burnout and the use of the MBI to assess it is appropriate.

For use by BENZAKOUR Lamyae only. Received from Mind Garden, Inc. on December 17, 2021

MBI Human Services Survey

Christina Maslach & Susan E. Jackson

The purpose of this survey is to discover how various people working in human services or the helping professions view their job and the people with whom they work closely.

Because people in a wide variety of occupations will answer this survey, it uses the term *recipients* to refer to the people for whom you provide your service, care, treatment, or instruction. When answering this survey please think of these people as recipients of the service you provide, even though you may use another term in your work.

Instructions: On the following page are 22 statements of job-related feelings. Please read each statement carefully and decide if you ever feel this way about *your* job. If you have *never* had this feeling, write the number "0" (zero) in the space before the statement. If you have had this feeling, indicate *how often* you feel it by writing the number (from 1 to 6) that best describes how frequently you feel that way. An example is shown below.

Example:

**How often:**	**0**	**1**	**2**	**3**	**4**	**5**	**6**
	Never	A few times a year or less	Once a month or less	A few times a month	Once a week	A few times a week	Every day

**How often 0-6**	**Statement:**
1. _________	I feel depressed at work.

If you never feel depressed at work, you would write the number "0" (zero) under the heading "How often." If you rarely feel depressed at work (a few times a year or less), you would write the number "1." If your feelings of depression are fairly frequent (a few times a week but not daily), you would write the number "5."

MBI - Human Services Survey - MBI-HSS: Copyright ©1981 Christina Maslach & Susan E. Jackson.
All rights reserved in all media. Published by Mind Garden, Inc., www.mindgarden.com

For use by BENZAKOUR Lamyae only. Received from Mind Garden, Inc. on December 17, 2021

MBI Human Services Survey

**How often:**	**0**	**1**	**2**	**3**	**4**	**5**	**6**
	Never	A few times a year or less	Once a month or less	A few times a month	Once a week	A few times a week	Every day

**How often 0-6**	**Statements:**
1. _________	I feel emotionally drained from my work.
2. _________	I feel used up at the end of the workday.
3. _________	I feel fatigued when I get up in the morning and have to face another day on the job.
4. _________	I can easily understand how my recipients feel about things.
5. _________	I feel I treat some recipients as if they were impersonal objects.
6. _________	Working with people all day is really a strain for me.
7. _________	I deal very effectively with the problems of my recipients.
8. _________	I feel burned out from my work.
9. _________	I feel I'm positively influencing other people's lives through my work.
10. _________	I've become more callous toward people since I took this job.
11. _________	I worry that this job is hardening me emotionally.
12. _________	I feel very energetic.
13. _________	I feel frustrated by my job.
14. _________	I feel I'm working too hard on my job.
15. _________	I don't really care what happens to some recipients.
16. _________	Working with people directly puts too much stress on me.
17. _________	I can easily create a relaxed atmosphere with my recipients.
18. _________	I feel exhilarated after working closely with my recipients.
19. _________	I have accomplished many worthwhile things in this job.
20. _________	I feel like I'm at the end of my rope.
21. _________	In my work, I deal with emotional problems very calmly.
22. _________	I feel recipients blame me for some of their problems.

(Administrative use only)

EE Total score: ________ DP Total score: ________ PA Total score: ________

EE Average score: ______ DP Average score: ______ PA Average score: ______

MBI - Human Services Survey - MBI-HSS: Copyright ©1981 Christina Maslach & Susan E. Jackson.
All rights reserved in all media. Published by Mind Garden, Inc., www.mindgarden.com

For use by BENZAKOUR Lamyae only. Received from Mind Garden, Inc. on December 17, 2021

MBI Human Services Survey for Medical Personnel

Christina Maslach & Susan E. Jackson

The purpose of this survey is to discover how various people in the human services or the helping professions view their job and the people with whom they work closely.

Instructions: On the following page are 22 statements of job-related feelings. Please read each statement carefully and decide if you ever feel this way about *your* job. If you have *never* had this feeling, write the number "0" (zero) in the space before the statement. If you have had this feeling, indicate *how often* you feel it by writing the number (from 1 to 6) that best describes how frequently you feel that way. An example is shown below.

Example:

**How often:**	**0**	**1**	**2**	**3**	**4**	**5**	**6**
	Never	A few times a year or less	Once a month or less	A few times a month	Once a week	A few times a week	Every day

**How often 0-6**	**Statement:**
1. _________	I feel depressed at work.

If you never feel depressed at work, you would write the number "0" (zero) under the heading "How often." If you rarely feel depressed at work (a few times a year or less), you would write the number "1." If your feelings of depression are fairly frequent (a few times a week but not daily), you would write the number "5."

MBI - Human Services Survey for Medical Personnel - MBI-HSS (MP): Copyright ©1981, 2016 Christina Maslach & Susan E. Jackson. All rights reserved in all media. Published by Mind Garden, Inc., www.mindgarden.com

For use by BENZAKOUR Lamyae only. Received from Mind Garden, Inc. on December 17, 2021

MBI Human Services Survey for Medical Personnel

How often:	0	1	2	3	4	5	6
	Never	A few times a year or less	Once a month or less	A few times a month	Once a week	A few times a week	Every day

How often 0-6		Statements:
1.	_________	I feel emotionally drained from my work.
2.	_________	I feel used up at the end of the workday.
3.	_________	I feel fatigued when I get up in the morning and have to face another day on the job.
4.	_________	I can easily understand how my patients feel about things.
5.	_________	I feel I treat some patients as if they were impersonal objects.
6.	_________	Working with people all day is really a strain for me.
7.	_________	I deal very effectively with the problems of my patients.
8.	_________	I feel burned out from my work.
9.	_________	I feel I'm positively influencing other people's lives through my work.
10.	_________	I've become more callous toward people since I took this job.
11.	_________	I worry that this job is hardening me emotionally.
12.	_________	I feel very energetic.
13.	_________	I feel frustrated by my job.
14.	_________	I feel I'm working too hard on my job.
15.	_________	I don't really care what happens to some patients.
16.	_________	Working with people directly puts too much stress on me.
17.	_________	I can easily create a relaxed atmosphere with my patients.
18.	_________	I feel exhilarated after working closely with my patients.
19.	_________	I have accomplished many worthwhile things in this job.
20.	_________	I feel like I'm at the end of my rope.
21.	_________	In my work, I deal with emotional problems very calmly.
22.	_________	I feel patients blame me for some of their problems.

(Administrative use only)

EE Total score: _________ DP Total score: _________ PA Total score: __________

EE Average score: ______ DP Average score: _______ PA Average score: _______

MBI - Human Services Survey for Medical Personnel - MBI-HSS (MP): Copyright ©1981, 2016 Christina Maslach & Susan E. Jackson. All rights reserved in all media. Published by Mind Garden, Inc., www.mindgarden.com

For use by BENZAKOUR Lamyae only. Received from Mind Garden, Inc. on December 17, 2021

MBI for Educators Survey

Christina Maslach, Susan E. Jackson & Richard L. Schwab

The purpose of this survey is to discover how educators view their job and the people with whom they work closely.

Instructions: On the following page are 22 statements of job-related feelings. Please read each statement carefully and decide if you ever feel this way about *your* job. If you have *never* had this feeling, write the number "0" (zero) in the space before the statement. If you have had this feeling, indicate *how often* you feel it by writing the number (from 1 to 6) that best describes how frequently you feel that way. An example is shown below.

**How often:**	**0**	**1**	**2**	**3**	**4**	**5**	**6**
	Never	A few times a year or less	Once a month or less	A few times a month	Once a week	A few times a week	Every day

Example:

**How often 0-6**	**Statement:**
1. _________	I feel depressed at work.

If you never feel depressed at work, you would write the number "0" (zero) under the heading "How often." If you rarely feel depressed at work (a few times a year or less), you would write the number "1." If your feelings of depression are fairly frequent (a few times a week but not daily), you would write the number "5."

MBI - Educators Survey - MBI-ES: Copyright ©1986 Christina Maslach, Susan E. Jackson & Richard L. Schwab. All rights reserved in all media. Published by Mind Garden, Inc., www.mindgarden.com

For use by BENZAKOUR Lamyae only. Received from Mind Garden, Inc. on December 17, 2021

MBI for Educators Survey

**How often:**	**0**	**1**	**2**	**3**	**4**	**5**	**6**
	Never	A few times a year or less	Once a month or less	A few times a month	Once a week	A few times a week	Every day

**How often 0-6**	**Statements:**
1. _________	I feel emotionally drained from my work.
2. _________	I feel used up at the end of the workday.
3. _________	I feel fatigued when I get up in the morning and have to face another day on the job.
4. _________	I can easily understand how my students feel about things.
5. _________	I feel I treat some students as if they were impersonal objects.
6. _________	Working with people all day is really a strain for me.
7. _________	I deal very effectively with the problems of my students.
8. _________	I feel burned out from my work.
9. _________	I feel I'm positively influencing other people's lives through my work.
10. _________	I've become more callous toward people since I took this job.
11. _________	I worry that this job is hardening me emotionally.
12. _________	I feel very energetic.
13. _________	I feel frustrated by my job.
14. _________	I feel I'm working too hard on my job.
15. _________	I don't really care what happens to some students.
16. _________	Working with people directly puts too much stress on me.
17. _________	I can easily create a relaxed atmosphere with my students.
18. _________	I feel exhilarated after working closely with my students.
19. _________	I have accomplished many worthwhile things in this job.
20. _________	I feel like I'm at the end of my rope.
21. _________	In my work, I deal with emotional problems very calmly.
22. _________	I feel students blame me for some of their problems.

(Administrative use only)

EE Total score: _________ DP Total score: _________ PA Total score: _________

EE Average score: ______ DP Average score: ______ PA Average score: _______

MBI - Educators Survey - MBI-ES: Copyright ©1986 Christina Maslach, Susan E. Jackson & Richard L. Schwab. All rights reserved in all media. Published by Mind Garden, Inc., www.mindgarden.com

For use by BENZAKOUR Lamyae only. Received from Mind Garden, Inc. on December 17, 2021

MBI – Human Services, Medical Personnel, and Educators Scoring Key
Emotional Exhaustion (EE) Subscale

Directions: Line up this scoring key with the MBI survey form. Sum the survey responses on EE items # 1, 2, 3, 6, 8, 13, 14, 16, and 20 that correspond to the unshaded areas on this scoring key. Enter this EE total score on the survey form. Divide the EE total score by the number of answered EE items for an EE average score. Research usually reports the average score.

How Often 0-6
1. _________
2. _________
3. _________
4. _________
5. _________
6. _________
7. _________
8. _________
9. _________
10. _________
11. _________
12. _________
13. _________
14. _________
15. _________
16. _________
17. _________
18. _________
19. _________
20. _________
21. _________
22. _________

MBI - Human Services Survey - MBI-HSS: Copyright ©1981 Christina Maslach & Susan E. Jackson.

MBI - Human Services Survey for Medical Personnel - MBI-HSS (MP): Copyright ©1981, 2016 by Christina Maslach & Susan E. Jackson.

MBI - Educators Survey - MBI-ES: Copyright ©1986 Christina Maslach, Susan E. Jackson & Richard L. Schwab. All rights reserved in all media. Published by Mind Garden, Inc., www.mindgarden.com

For use by BENZAKOUR Lamyae only. Received from Mind Garden, Inc. on December 17, 2021

MBI – Human Services, Medical Personnel, and Educators Scoring Key Depersonalization (DP) Subscale

Directions: Line up this scoring key with the MBI survey form. Sum the survey responses on DP items # 5, 10, 11, 15, and 22 that correspond to the unshaded areas on this scoring key. Enter this DP total score on the survey form. Divide the DP total score by the number of answered DP items for a DP average score. Research usually reports the average score.

How Often 0-6
1. _________
2. _________
3. _________
4. _________
5. _________
6. _________
7. _________
8. _________
9. _________
10. _________
11. _________
12. _________
13. _________
14. _________
15. _________
16. _________
17. _________
18. _________
19. _________
20. _________
21. _________
22. _________

MBI – Human Services Survey - MBI-HSS: Copyright ©1981 Christina Maslach & Susan E. Jackson.

MBI - Human Services Survey for Medical Personnel - MBI-HSS (MP): Copyright ©1981, 2016 by Christina Maslach & Susan E. Jackson.

MBI - Educators Survey - MBI-ES: Copyright ©1986 Christina Maslach, Susan E. Jackson & Richard L. Schwab. All rights reserved in all media. Published by Mind Garden, Inc., www.mindgarden.com

For use by BENZAKOUR Lamyae only. Received from Mind Garden, Inc. on December 17, 2021

MBI – Human Services, Medical Personnel, and Educators Scoring Key
Personal Accomplishment (PA) Subscale

Directions: Line up this scoring key with the MBI survey form. Sum the survey responses on PA items # 4, 7, 9, 12, 17, 18, 19, and 21 that correspond to the unshaded areas on this scoring key. Enter this PA total score on the survey form. Divide the PA total score by the number of answered PA items for a PA average score. Research usually reports the average score.

How Often 0-6
1. _________
2. _________
3. _________
4. _________
5. _________
6. _________
7. _________
8. _________
9. _________
10. _________
11. _________
12. _________
13. _________
14. _________
15. _________
16. _________
17. _________
18. _________
19. _________
20. _________
21. _________
22. _________

MBI – Human Services Survey - MBI-HSS: Copyright ©1981 Christina Maslach & Susan E. Jackson.

MBI - Human Services Survey for Medical Personnel - MBI-HSS (MP): Copyright ©1981, 2016 by Christina Maslach & Susan E. Jackson.

MBI - Educators Survey - MBI-ES: Copyright ©1986 Christina Maslach, Susan E. Jackson & Richard L. Schwab. All rights reserved in all media. Published by Mind Garden, Inc., www.mindgarden.com

For use by BENZAKOUR Lamyae only. Received from Mind Garden, Inc. on December 17, 2021

MBI - General Survey

Wilmar B. Schaufeli, Michael P. Leiter, Christina Maslach & Susan E. Jackson

The purpose of this survey is to discover how staff members view their job, and their reactions to their work.

Instructions: On the following page are 16 statements of job-related feelings. Please read each statement carefully and decide if you ever feel this way about *your* job. If you have *never* had this feeling, write the number “0” (zero) in the space before the statement. If you have had this feeling, indicate *how often* you feel it by writing the number (from 1 to 6) that best describes how frequently you feel that way. An example is shown below.

Example:

**How often:**	**0**	**1**	**2**	**3**	**4**	**5**	**6**
	Never	A few times a year or less	Once a month or less	A few times a month	Once a week	A few times a week	Every day

**How often 0-6**	**Statement:**
1. _________	I feel depressed at work.

If you never feel depressed at work, you would write the number “0” (zero) under the heading “How often.” If you rarely feel depressed at work (a few times a year or less), you would write the number “1.” If your feelings of depression are fairly frequent (a few times a week but not daily), you would write the number “5.”

MBI - General Survey - MBI-GS: Copyright ©1996 Wilmar B. Schaufeli, Michael P. Leiter, Christina Maslach & Susan E. Jackson & Richard L. Schwab. All rights reserved in all media. Published by Mind Garden, Inc., www.mindgarden.com

For use by BENZAKOUR Lamyae only. Received from Mind Garden, Inc. on December 17, 2021

MBI - General Survey

**How often:**	**0**	**1**	**2**	**3**	**4**	**5**	**6**
	Never	A few times a year or less	Once a month or less	A few times a month	Once a week	A few times a week	Every day

**How often 0-6**	**Statements:**
1. ________	I feel emotionally drained from my work.
2. ________	I feel used up at the end of the workday.
3. ________	I feel tired when I get up in the morning and have to face another day on the job.
4. ________	Working all day is really a strain for me.
5. ________	I can effectively solve the problems that arise in my work.
6. ________	I feel burned out from my work.
7. ________	I feel I am making an effective contribution to what this organization does.
8. ________	I have become less interested in my work since I started this job.
9. ________	I have become less enthusiastic about my work.
10. ________	In my opinion, I am good at my job.
11. ________	I feel exhilarated when I accomplish something at work.
12. ________	I have accomplished many worthwhile things in this job.
13. ________	I just want to do my job and not be bothered.
14. ________	I have become more cynical about whether my work contributes anything.
15. ________	I doubt the significance of my work.
16. ________	At my work, I feel confident that I am effective at getting things done.

(Administrative use only)

EX Total score: ________ CY Total score: ________ PE Total score: _________

EX Average score: ______ CY Average score: ______ PE Average score:_______

MBI - General Survey - MBI-GS: Copyright ©1996 Wilmar B. Schaufeli, Michael P. Leiter, Christina Maslach & Susan E. Jackson & Richard L. Schwab. All rights reserved in all media. Published by Mind Garden, Inc., www.mindgarden.com

For use by BENZAKOUR Lamyae only. Received from Mind Garden, Inc. on December 17, 2021

MBI - General Survey for Students

Wilmar B. Schaufeli, Michael P. Leiter, Christina Maslach & Susan E. Jackson

The purpose of this survey is to discover how university students view their studies, and their reactions to their academic work.

Instructions: On the following page are 16 statements of university-related feelings. Please read each statement carefully and decide if you ever feel this way about *your* academic work. If you have *never* had this feeling, write the number "0" (zero) in the space before the statement. If you have had this feeling, indicate *how often* you feel it by writing the number (from 1 to 6) that best describes how frequently you feel that way. An example is shown below.

Example:

**How often:**	**0**	**1**	**2**	**3**	**4**	**5**	**6**
	Never	A few times a year or less	Once a month or less	A few times a month	Once a week	A few times a week	Every day

**How often 0-6**	**Statement:**
1. _________	I feel depressed by my studies.

If you never feel depressed by your studies, you would write the number "0" (zero) under the heading "How often." If you rarely feel depressed by your studies, (a few times a year or less), you would write the number "1." If your feelings of being depressed by your studies are fairly frequent (a few times a week but not daily), you would write the number "5."

MBI - General Survey for Students – MBI-GS (S): Copyright ©1996, 2016 Wilmar B. Schaufeli, Michael P. Leiter, Christina Maslach & Susan E. Jackson. All rights reserved in all media. Published by Mind Garden, Inc. www.mindgarden.com.

For use by BENZAKOUR Lamyae only. Received from Mind Garden, Inc. on December 17, 2021

MBI - General Survey for Students

**How often:**	**0**	**1**	**2**	**3**	**4**	**5**	**6**
	Never	A few times a year or less	Once a month or less	A few times a month	Once a week	A few times a week	Every day

**How Often 0-6**	**Statements:**
1. ______	I feel emotionally drained by my studies.
2. ______	I feel used up at the end of the day at the university.
3. ______	I feel tired when I get up in the morning and have to face another day at the university.
4. ______	Attending classes all day is really a strain for me.
5. ______	I can effectively solve the problems that arise in my studies.
6. ______	I feel burned out from my studies.
7. ______	I feel I am making an effective contribution in class.
8. ______	I have become less interested in my studies since my enrollment.
9. ______	I have become less enthusiastic about my studies.
10. ______	In my opinion, I am a good student.
11. ______	I feel exhilarated when I accomplish something at the university.
12. ______	I have accomplished many worthwhile things in my studies.
13. ______	I just want to get my work done and not be bothered.
14. ______	I have become more cynical about whether my university work contributes anything.
15. ______	I doubt the significance of my studies.
16. ______	While working at the university, I feel confident that I am effective at getting things done.

(Administrative use only)

EX Total score: ________ CY Total score: ________ PE Total score: _________

EX Average score: ______ CY Average score: ______ PE Average score: ______

MBI - General Survey for Students – MBI-GS (S): Copyright ©1996, 2016 Wilmar B. Schaufeli, Michael P. Leiter, Christina Maslach & Susan E. Jackson. All rights reserved in all media. Published by Mind Garden, Inc. www.mindgarden.com.

For use by BENZAKOUR Lamyae only. Received from Mind Garden, Inc. on December 17, 2021

MBI – General Survey and General Survey for Students Scoring Key Exhaustion (EX) Subscale

Directions: Line up this scoring key with the MBI survey form. Sum the survey responses on EX items # 1, 2, 3, 4, and 6 that correspond to the unshaded areas on this scoring key. Enter this EX total score on the survey form. Divide the EX total score by the number of answered EX items for an EX average score. Research usually reports the average score.

How Often 0-6
1. _________
2. _________
3. _________
4. _________
5. _________
6. _________
7. _________
8. _________
9. _________
10. _________
11. _________
12. _________
13. _________
14. _________
15. _________
16. _________

MBI - General Survey – MBI-GS: Copyright ©1996 Wilmar B. Schaufeli, Michael P. Leiter, Christina Maslach & Susan E. Jackson.

MBI - General Survey for Students – MBI-GS (S): Copyright ©1996, 2016 Wilmar B. Schaufeli, Michael P. Leiter, Christina Maslach & Susan E. Jackson.

All rights reserved in all media. Published by Mind Garden, Inc. www.mindgarden.com.

For use by BENZAKOUR Lamyae only. Received from Mind Garden, Inc. on December 17, 2021

MBI – General Survey and General Survey for Students Scoring Key
Cynicism (CY) Subscale

Directions: Line up this scoring key with the MBI survey form. Sum the survey responses on CY items # 8, 9, 13, 14, and 15 that correspond to the unshaded areas on this scoring key. Enter this CY total score on the survey form. Divide the CY total score by the number of answered CY items for a CY average score. Research usually reports the average score.

How Often 0-6
1. _________
2. _________
3. _________
4. _________
5. _________
6. _________
7. _________
8. _________
9. _________
10. _________
11. _________
12. _________
13. _________
14. _________
15. _________
16. _________

MBI - General Survey – MBI-GS: Copyright ©1996 Wilmar B. Schaufeli, Michael P. Leiter, Christina Maslach & Susan E. Jackson.

MBI - General Survey for Students – MBI-GS (S): Copyright ©1996, 2016 Wilmar B. Schaufeli, Michael P. Leiter, Christina Maslach & Susan E. Jackson.

All rights reserved in all media. Published by Mind Garden, Inc. www.mindgarden.com.

For use by BENZAKOUR Lamyae only. Received from Mind Garden, Inc. on December 17, 2021

MBI – General Survey and General Survey for Students Scoring Key Professional Efficacy (PE) Subscale

Directions: Line up this scoring key with the MBI survey form. Sum the survey responses on PE items # 5, 7, 10, 11, 12, and 16 that correspond to the unshaded areas on this scoring key. Enter this PE total score on the survey form. Divide the PE total score by the number of answered PE items for a PE average score. Research usually reports the average score.

	How Often 0-6
1.	_________
2.	_________
3.	_________
4.	_________
5.	_________
6.	_________
7.	_________
8.	_________
9.	_________
10.	_________
11.	_________
12.	_________
13.	_________
14.	_________
15.	_________
16.	_________

MBI - General Survey – MBI-GS: Copyright ©1996 Wilmar B. Schaufeli, Michael P. Leiter, Christina Maslach & Susan E. Jackson.

MBI - General Survey for Students – MBI-GS (S): Copyright ©1996, 2016 Wilmar B. Schaufeli, Michael P. Leiter, Christina Maslach & Susan E. Jackson.

All rights reserved in all media. Published by Mind Garden, Inc. www.mindgarden.com.

Indiquez la fréquence à laquelle vous ressentez ce qui est décrit à chaque phrase en encerclant le chiffre correspondant. Si vous ne le ressentez pas du tout, encerclez le chiffre "0" et continuez avec la phrase suivante.

**Fréquence :**	jamais	quelques fois par année ou moins	une fois par mois ou moins	quelques fois par mois	une fois par semaine	quelques fois par semaine	chaque jour
	0	1	2	3	4	5	6

	**Fréquence**						
1. Je me sens émotionnellement accaparé(e) par mon travail.	0	1	2	3	4	5	6
2. Je me sens à bout à la fin d'une journée de travail.	0	1	2	3	4	5	6
3. Je me sens fatigué(e) lorsque je me lève le matin et que j'ai à affronter une autre journée de travail.	0	1	2	3	4	5	6
4. Je peux comprendre facilement ce que mes patients ressentent.	0	1	2	3	4	5	6
5. Je sens que je traite certains patients de façon impersonnelle, comme s'ils étaient des objets.	0	1	2	3	4	5	6
6. Travailler chaque jour avec des patients, c'est vraiment un fardeau pour moi.	0	1	2	3	4	5	6
7. Je prends en charge avec efficacité les problèmes de mes patients.	0	1	2	3	4	5	6
8. Je me sens épuisé(e) psychologiquement par à mon travail.	0	1	2	3	4	5	6
9. Je pense que, grâce à mon travail j'ai une influence positive sur la vie de mes patients.	0	1	2	3	4	5	6
10. Je suis devenu(e) plus dur(e) envers les gens depuis que j'ai cet emploi.	0	1	2	3	4	5	6
11. Je crains que ce travail ne me rende moins sensible.	0	1	2	3	4	5	6
12. Je me sens très énergique.	0	1	2	3	4	5	6
13. Je me sens frustré(e) par mon travail.	0	1	2	3	4	5	6
14. Je sens que je travaille trop durement à mon emploi.	0	1	2	3	4	5	6
15. Je ne me fais pas vraiment de souci pour ce qui arrive à quelques-uns de mes patients.	0	1	2	3	4	5	6
16. Travailler directement avec des patients me stresse trop.	0	1	2	3	4	5	6
17. Je peux facilement créer une atmosphère détendue avec mes patients.	0	1	2	3	4	5	6
18. Je me sens épanoui(e) lorsque j'ai travaillé étroitement avec mes patients.	0	1	2	3	4	5	6
19. J'ai accompli beaucoup de choses utiles dans ce travail.	0	1	2	3	4	5	6
20. Je me sens comme si j'étais au bout du rouleau.	0	1	2	3	4	5	6
21. Dans mon travail, je traite les problèmes émotionnels très calmement.	0	1	2	3	4	5	6
22. Je sens que mes patients me reprochent certains de leurs problèmes.	0	1	2	3	4	5	6

Research Edition Translation performed by U. Scherer, Ph.D. & D. Smaga, M.D., University of Geneva, on this date Aug. 1, 1987. Translated and reproduced by special permission of the Publisher, Consulting Psychologists Press, Inc., Palo Alto, CA 94303 from **Maslach Burnout-Inventory - Human Services Survey** by Christina Maslach and Susan E. Jackson. Copyright 1986 by Consulting Psychologists Press, Inc. Further reproduction is prohited without the Publisher's written consent.

	Jamais	Quelques fois par an au maximum	Une fois par mois au maximum	Plusieurs fois par mois	Une fois par semaine	Plusieurs fois par semaine	Tous les jours
1. Je me sens envahi émotionnellement par mon travail.	0☐	1☐	2☐	3☐	4☐	5☐	6☐
2. Je me sens épuisé à la fin de ma journée de travail.	0☐	1☐	2☐	3☐	4☐	5☐	6☐
3. Je me sens fatigué quand je me lève le matin et que je dois faire face à une autre journée de travail.	0☐	1☐	2☐	3☐	4☐	5☐	6☐
4. Je peux facilement comprendre comment mes patients ressentent les choses.	0☐	1☐	2☐	3☐	4☐	5☐	6☐
5. J'ai l'impression que je soigne des patients comme s'ils étaient des objets et non des personnes.	0☐	1☐	2☐	3☐	4☐	5☐	6☐
6. Travailler avec des personnes toute la journée est vraiment une contrainte pour moi.	0☐	1☐	2☐	3☐	4☐	5☐	6☐
7. Je traite efficacement les problèmes de mes patients.	0☐	1☐	2☐	3☐	4☐	5☐	6☐
8. Je me sens totalement épuisé par mon travail.	0☐	1☐	2☐	3☐	4☐	5☐	6☐
9. Je sens que j'influence vraiment la vie des autres personnes.	0☐	1☐	2☐	3☐	4☐	5☐	6☐
10. Je suis devenu plus distant avec les gens depuis que j'ai commencé ce travail.	0☐	1☐	2☐	3☐	4☐	5☐	6☐
11. Je crains que ce travail endurcisse mes émotions.	0☐	1☐	2☐	3☐	4☐	5☐	6☐
12. Je me sens plein d'énergie.	0☐	1☐	2☐	3☐	4☐	5☐	6☐
13. Je me sens frustré par mon travail.	0☐	1☐	2☐	3☐	4☐	5☐	6☐
14. Je trouve que je travaille trop dans mon travail	0☐	1☐	2☐	3☐	4☐	5☐	6☐
15. Je ne m'inquiète pas vraiment de ce qui arrive à certains patients.	0☐	1☐	2☐	3☐	4☐	5☐	6☐
16. Travailler directement avec des personnes m'occasionne trop de stress.	0☐	1☐	2☐	3☐	4☐	5☐	6☐
17. Je peux facilement créer une atmosphère de tranquillité avec mes patients.	0☐	1☐	2☐	3☐	4☐	5☐	6☐
18. J'accomplis beaucoup de choses valables dans ce travail.	0☐	1☐	2☐	3☐	4☐	5☐	6☐
19. Je me sens vivifié après avoir travaillé étroitement avec mes patients.	0☐	1☐	2☐	3☐	4☐	5☐	6☐
20. Je me sens au bout du rouleau.	0☐	1☐	2☐	3☐	4☐	5☐	6☐
21. Dans mon travail, je traite très calmement les problèmes émotionnels.	0☐	1☐	2☐	3☐	4☐	5☐	6☐
22. Je sens que des patients me reprochent certains de leurs problèmes.	0☐	1☐	2☐	3☐	4☐	5☐	6☐

Research Edition Translation performed by Olivier Thonon & Sabine Stordeur on March 25, 2009. Translated and reproduced by special permission of the Publisher, CPP, Inc., Mountain View, CA 94043 from Maslach Burnout Inventory-HSS by Christina Maslach, and Susan E. Jackson. Copyright 1985 by CPP, Inc. All rights reserved. Further reproduction is prohibited without the Publisher's written consent.

MBI - Human Services Survey - MBI-HSS: Copyright ©1981 Christina Maslach & Susan E. Jackson.
All rights reserved in all media. Published by Mind Garden, Inc., www.mindgarden.com

For use by BENZAKOUR Lamyae only. Received from Mind Garden, Inc. on December 17, 2021

Les déclarations qui suivent portent sur votre expérience professionnelle. *Veuillez accorder à chaque affirmation la valeur (chiffre allant de 0 à 6) qui correspond le mieux à votre perception. Les chiffres correspondent aux valeurs suivantes*

0 =	*1 =*	*2 =*	*3 =*	*4 =*	*5 =*	*6 =*
jamais	*sporadiquement quelques fois par an ou moins*	*de temps en temps, une fois par mois ou moins*	*régulièrement, quelques fois par mois*	*souvent, une fois par semaine*	*très souvent, plusieurs fois par semaine*	*toujours, chaque jour*

	Jamais	sporadique	De temps en temps	Régulièrement	Souvent	Tres souvent	Toujours
1. Je me sens émotionnellement épuisé(e) par mon travail	0	1	2	3	4	5	6
2. Je me sens vidé(e) à la fin d'une journée de travail	0	1	2	3	4	5	6
3. Je ressens de la fatigue en envisageant une nouvelle journée de travail	0	1	2	3	4	5	6
4. Je peux facilement comprendre le ressenti de mes usagers	0	1	2	3	4	5	6
5. J'ai le sentiment que je traite certains usagers de manière trop impersonnelle	0	1	2	3	4	5	6
6. Être en contact toute la journée avec d'autres personnes est une charge de travail	0	1	2	3	4	5	6
7. Je sais prendre en charge les problèmes de mes usagers de manière efficace	0	1	2	3	4	5	6
8. Je me sens épuisé(e) par mon travail	0	1	2	3	4	5	6
9. J'ai l'impression que j'influence positivement la vie des autres par mon travail	0	1	2	3	4	5	6
10. J'ai l'impression que je suis devenu(e) plus indifférent(e) à l'égard des autres personnes depuis que j'exerce ce travail	0	1	2	3	4	5	6
11. Je crains que mon travail ne me rende plus dur, émotionnellement, plus insensible	0	1	2	3	4	5	6
12. Je me sens plein(e) d'énergie	0	1	2	3	4	5	6
13. Je me sens frustré(e) par mon travail	0	1	2	3	4	5	6
14. Je pense que je m'investis trop dans mon travail	0	1	2	3	4	5	6

MBI-General Survey: Copyright ©1996 Wilmar B. Schaufeli, Michael P. Leiter, Christina Maslach & Susan E. Jackson.
MBI-Human Services Survey: Copyright ©1981 Christina Maslach & Susan E. Jackson.
MBI-Educators Survey: Copyright ©1986 Christina Maslach, Susan E. Jackson & Richard L. Schwab.
All rights reserved in all media. Published by Mind Garden, Inc., www.mindgarden.com

For use by BENZAKOUR Lamyae only. Received from Mind Garden, Inc. on December 17, 2021

15. Ce qui arrive à certains usagers me laisse indifférent(e)	0	1	2	3	4	5	6
16. Travailler avec d’autres personnes me stresse trop	0	1	2	3	4	5	6
17. Je peux facilement créer une atmosphère détendue avec mes usagers	0	1	2	3	4	5	6
18. Travailler avec des usagers me stimule	0	1	2	3	4	5	6
19. J’ai accompli beaucoup de choses intéressantes dans le cadre de ce travail	0	1	2	3	4	5	6
20. Je me sens au bout du rouleau	0	1	2	3	4	5	6
21. Je gère calmement les problèmes émotionnels dans mon travail	0	1	2	3	4	5	6
22. J’ai le sentiment que certains usagers me reprochent leurs problèmes	0	1	2	3	4	5	6

MBI-General Survey: Copyright ©1996 Wilmar B. Schaufeli, Michael P. Leiter, Christina Maslach & Susan E. Jackson.
MBI-Human Services Survey: Copyright ©1981 Christina Maslach & Susan E. Jackson.
MBI-Educators Survey: Copyright ©1986 Christina Maslach, Susan E. Jackson & Richard L. Schwab.
All rights reserved in all media. Published by Mind Garden, Inc., www.mindgarden.com

For use by BENZAKOUR Lamyae only. Received from Mind Garden, Inc. on December 17, 2021

Jamais	Quelques fois par année ou moins	Une fois par mois ou moins	Quelques fois par mois	Une fois par semaine	Quelques fois par semaine	Chaque jour
0	1	2	3	4	5	6

1. Je me sens émotionnellement vidé(e) par mon travail.
2. Je suis exténué(e) à la fin de ma journée de travail.
3. Lorsque je me réveille, je me sens fatigué(e) à l'idée d'avoir à faire face à une autre journée de travail.
4. Travailler toute la journée est vraiment éprouvant pour moi.
5. Je suis capable de résoudre de façon efficace les problèmes que je rencontre dans mon travail.
6. Je me sens épuisé(e) par mon travail.
7. J'estime que j'apporte une véritable contribution au sein de mon entreprise/institution.
8. Je suis moins intéressé(e) par mon travail que je ne l'étais au début.
9. Mon travail m'enthousiasme moins qu'avant.
10. De mon point de vue, je fais du bon travail.
11. Je me sens stimulé(e) quand je mène à bien une tâche professionnelle.
12. J'ai accompli plusieurs choses utiles à ce poste.
13. Au travail, je veux juste faire ce qui est attendu de moi et rien de plus.
14. Je suis devenu(e) plus sceptique quant à l'utilité de mon travail.
15. Je doute de l'importance du travail que j'effectue.
16. Dans mon travail, j'ai confiance en ma capacité à faire avancer les choses efficacement.

TA-675 – MBI-GS – French

MBI - General Survey - MBI-GS: Copyright ©1996 Wilmar B. Schaufeli, Michael P. Leiter, Christina Maslach & Susan E. Jackson & Richard L. Schwab. All rights reserved in all media. Published by Mind Garden, Inc., www.mindgarden.com

For use by BENZAKOUR Lamyae only. Received from Mind Garden, Inc. on December 17, 2021

For Publications:
We understand situations exist where you may want sample test questions for various fair use situations such as academic, scientific or commentary purposes. No items from this instrument may be included in any publication without the prior express written permission from Mind Garden, Inc. Please understand that disclosing more than we have authorized will compromise the integrity and value of the test.

For Dissertation and Thesis Appendices:
You may not include an entire instrument in your thesis or dissertation, however you may use the three sample items specified by Mind Garden. Academic committees understand the requirements of copyright and are satisfied with sample items for appendices and tables. For customers needing permission to reproduce the three sample items in a thesis or dissertation, the following page includes the permission letter and reference information needed to satisfy the requirements of an academic committee.

Online Use of Mind Garden Instruments:
Online administration and scoring of the Maslach Burnout Inventory is available from Mind Garden, (https://www.mindgarden.com/117-maslach-burnout-inventory). Mind Garden provides services to add items and demographics to the Maslach Burnout Inventory. Reports are available for the Maslach Burnout Inventory.

If your research uses an online survey platform other than the Mind Garden Transform survey system, you will need to meet Mind Garden’s requirements by following the procedure described at mindgarden.com/mind-garden-forms/58-remote-online-use-application.html.

All Other Special Reproductions:
For any other special purposes requiring permissions for reproduction of this instrument, please contact info@mindgarden.com.

For use by BENZAKOUR Lamyae only. Received from Mind Garden, Inc. on December 17, 2021

mind garden

www.mindgarden.com

To Whom It May Concern,

The above-named person has made a license purchase from Mind Garden, Inc. and has permission to administer the following copyrighted instrument up to that quantity purchased:

Maslach Burnout Inventory forms: Human Services Survey, Human Services Survey for Medical Personnel, Educators Survey, General Survey, or General Survey for Students.

The three sample items only from this instrument as specified below may be included in your thesis or dissertation. Any other use must receive prior written permission from Mind Garden. The entire instrument form may not be included or reproduced at any time in any other published material. Please understand that disclosing more than we have authorized will compromise the integrity and value of the test.

Citation of the instrument must include the applicable copyright statement listed below.
Sample Items:

MBI - Human Services Survey - MBI-HSS:

I feel emotionally drained from my work.
I have accomplished many worthwhile things in this job.
I don't really care what happens to some recipients.

Copyright ©1981 Christina Maslach & Susan E. Jackson. All rights reserved in all media. Published by Mind Garden, Inc., www.mindgarden.com

MBI - Human Services Survey for Medical Personnel - MBI-HSS (MP):

I feel emotionally drained from my work.
I have accomplished many worthwhile things in this job.
I don't really care what happens to some patients.

Copyright ©1981, 2016 by Christina Maslach & Susan E. Jackson. All rights reserved in all media. Published by Mind Garden, Inc., www.mindgarden.com

MBI - Educators Survey - MBI-ES:

I feel emotionally drained from my work.
I have accomplished many worthwhile things in this job.
I don't really care what happens to some students.

Copyright ©1986 Christina Maslach, Susan E. Jackson & Richard L. Schwab. All rights reserved in all media. Published by Mind Garden, Inc., www.mindgarden.com

Cont'd on next page

For use by BENZAKOUR Lamyae only. Received from Mind Garden, Inc. on December 17, 2021

MBI - General Survey - MBI-GS:

I feel emotionally drained from my work.
In my opinion, I am good at my job.
I doubt the significance of my work.

Copyright ©1996 Wilmar B. Schaufeli, Michael P. Leiter, Christina Maslach & Susan E. Jackson. All rights reserved in all media. Published by Mind Garden, Inc., www.mindgarden.com

MBI - General Survey for Students - MBI-GS (S):

I feel emotionally drained by my studies.
In my opinion, I am a good student.
I doubt the significance of my studies.

Copyright ©1996, 2016 Wilmar B. Schaufeli, Michael P. Leiter, Christina Maslach & Susan E. Jackson. All rights reserved in all media. Published by Mind Garden, Inc., www.mindgarden.com

Sincerely,

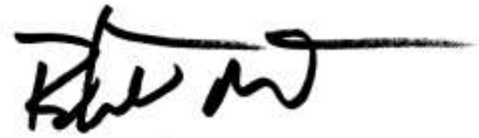

Robert Most
Mind Garden, Inc.
www.mindgarden.com
